# Why do pregnant women with fear of birth prefer vaginal birth? A qualitative study in China

**DOI:** 10.3389/fpsyg.2023.1110116

**Published:** 2023-02-23

**Authors:** Tieying Zeng, Mengmei Yuan, Meiliyang Wu, Ye Chen, Ke Zhang

**Affiliations:** ^1^Department of Nursing, Tongji Hospital, Tongji Medical College, Huazhong University of Science and Technology, Wuhan, China; ^2^School of Nursing, Tongji Medical College, Huazhong University of Science and Technology, Wuhan, China

**Keywords:** fear of birth, vaginal birth, pregnant women, qualitative study, culture

## Abstract

**Background:**

Fear of birth (FOB) is becoming increasingly recognized as a mental health issue that may endanger maternal and infant health and affects women’s subsequent fertility desires. It has also been shown to be related to the choice of delivery mode. Given the differences in healthcare systems and policies between countries, and the gaps in the exploration of women’s experience of fear of birth and its association with the delivery mode in the Chinese cultural context, this study thus attempt to understand Chinese women’s experience with fear of birth and their preferences for delivery mode through a qualitative study.

**Methods:**

A descriptive qualitative research was performed among twenty pregnant women from the obstetric outpatient of a tertiary hospital in China. Colaizzi’s method was used to analyze the participants’ data.

**Results:**

We proposed three themes and nine subthemes on the participants’ experience with fear of birth: (1) an invisible dilemma: trapped in lingering fear (fear of all sides, the untold loneliness, and ambivalence with mixed feelings); (2) an unexpected decision: choose to give birth naturally (initiative selection and passive acceptance); and (3) A strength to confront challenges head-on: move forward with fear (awaken of maternal spirit, hope in bloom, Chinese tolerance culture, and obstetric analgesia).

**Conclusion:**

Fear of birth is a complex emotion, accompanied by feelings of loneliness and ambivalence in addition to fear. We found that women with fear of birth in this study prefer vaginal birth, and it was revealed to be the result of a combined action of intrinsic and extrinsic factors.

## Introduction

Fear of birth (FOB) is becoming increasingly recognized as a mental health issue, which is manifested as nightmares, physical complaints, and difficulties in concentrating on work or on family activities (Nilsson et al., 2018). FOB is a common phenomenon in pregnant women and is widely distributed around the world. A meta-analysis reported that the global pooled prevalence at 14%, and the prevalence varies between countries, from 3.7–43%. FOB exists on a continuum, from normal worry to severe fear of birth (toco-phobia) (O’Connell et al., 2017), and can be experienced during pregnancy, during labor, and after delivery. Previous studies have reported that fear and its associated stress can also cause more serious problems that may contribute to premature delivery, obstetric complications, adverse birth outcomes, and a higher risk for postpartum depression and postanal traumatic stress disorder (PTSD) (Nilsson et al., 2012; Karlström et al., 2013). What’s more, frightened women are inclined to have a negative and traumatic experience, which has been reported to significantly interfere with the partner, family, and mother-infant relationship and affect women’s subsequent fertility desires (Nicholls and Ayers, 2007).

Given the harm caused by fear of birth, it is currently being explored in depth worldwide. Numerous qualitative studies have stated that fear of birth is multi-dimensional (Nystedt et al., 2014; Russo et al., 2015). In addition to fear of labor pain, fear of birth includes fear of new-born health, fear of losing control during labor, and fear of medical intervention or the hospital environment. Moreover, researchers have demonstrated significant regional differences in women’s fears of childbirth, in which cultural context and variations in beliefs about childbirth play an important role (Callister and Khalaf, 2010; Russo et al., 2015). Factors such as medical environment, medical service, and community participation in different areas can also have implications for women’s fears (Takegata et al., 2018). Chinese unique cultural perspectives may affect women’s perceptions of childbirth. It is therefore vital to understand the fear of birth in the context of Chinese culture. However, the current research on the fear of birth in China has mainly focused on the level of childbirth fear in Chinese women and its influence factors through cross-sectional studies, while there has been a lack of in-depth exploration of the experience of fear of birth.

Fear of birth has been shown to be associated with the choice of delivery mode. Many studies in Western countries identified fear of birth as a highlighted reason for unnecessary cesarean sections without medical indication (Faisal et al., 2014; Coates et al., 2020). On the contrary, a Japanese qualitative study reported different results, in which 11 primiparas with fear of birth all stated in the interviews that they preferred vaginal birth unless the labor was abnormal (Takegata et al., 2018). But the researchers did not explain the reason for the preference. Notably, a study found that delivery-related control beliefs may play an important role in the relationship between fear of childbirth and delivery mode, as it was an important psychological characteristic in the prediction of preferences for delivery mode (Martos et al., 2021). Currently, China still has a high cesarean section rate of 41.5%, of which selective cesarean section accounts for 10.7% (Ming et al., 2019). Although there is a downward trend in recent years, it still is considerably greater than the level of 10–15%, which is recommended by the World Health Organization to reduce maternal and perinatal mortality rates (World Health Organization, 1985; Ye et al., 2016). Understanding the relationship between Chinese women’s fear of birth and delivery mode may help reduce the rate of elective C-sections. However, it is still unknown due to the lack of relevant research.

Given the dangers of childbirth fear to maternal and infant health, the differences in healthcare systems and policies between countries, and the gaps in the exploration of women’s experience of fear of birth and its association with the delivery mode in the Chinese cultural context, in this study, we thus attempt to understand pregnant women’s experience with fear of birth in the Chinese cultural context and their preferences for delivery mode through a qualitative study. This may contribute to identifying factors that can be intervened and developing antenatal interventions to reduce cesarean section rates. The research questions were:

• What are the pregnant women’s experiences with fear of birth in Chinese cultural context?
• What are the perceptions and preferences for delivery mode of Chinese pregnant women with fear of birth?

## Methods

### Study design

A descriptive qualitative design with in-depth interviews was utilized, as it was deemed appropriate for exploring the real and comprehensive feelings, attitudes, perceptions, and behaviors in the context of where the action occurred (Gerrish et al., 2015).

### Participant

In this study, pregnant women who underwent routine outpatient obstetric check-ups at a tertiary hospital in Wuhan, Hubei province between March 2019 and October 2020 were selected as the study population using a purposive sampling method. Subjects of different ages, occupations, education levels, parity, and gestational weeks were selected to obtain a more representative and diverse sample. The sample size was determined based on the criterion that the data of the respondents were repeated and no new themes were presented at the time of data analysis (data saturation). Those under 18 or with serious pregnancy complications (such as gestational hypertension, preeclampsia and premature rupture of membranes, etc.) were excluded. A total of 20 pregnant women were eventually enrolled in the study, 14 of whom were interviewed before the COVID-19 period and six were interviewed after the pandemic had been controlled.

### Data collection

When pregnant women came to the target hospital for routine outpatient obstetric check-ups, the researchers communicated with them while they were waiting for the examinations to know their general condition. For pregnant women who met the inclusion criteria, the researchers would inform them of the purpose, method, content, time of the interview, and recording requirements of the study, and those who agreed would be invited by the researchers to participate in the study. After the participants have completed their obstetric check-ups, the researcher would bring them to the obstetric classroom task room of the maternity clinic, which was chosen to eliminate unfamiliarity. Consenting participants were required to sign an informed consent form detailing the purpose of the study, participants’ rights, and guarantees of confidentiality and anonymity. During the interview, the environment was kept quiet and comfortable, and no extraneous people were allowed in or out to ensure the privacy of the interview.

**FIGURE 1:**
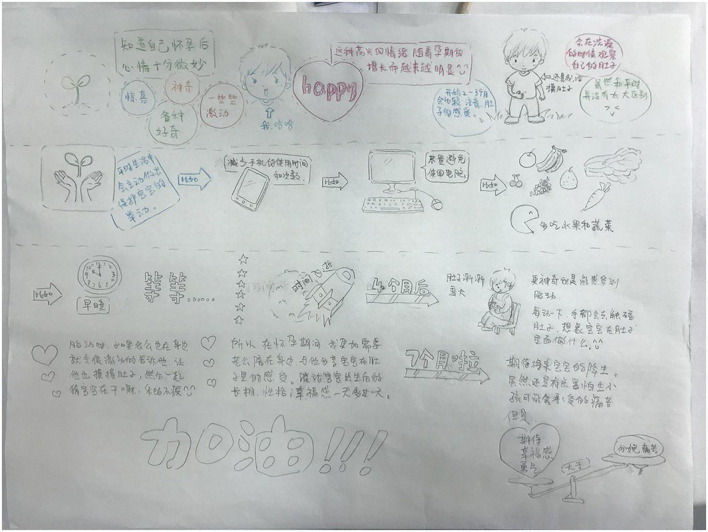
A painting showing the whole pregnancy experience and emotional journey.

**FIGURE 2:**
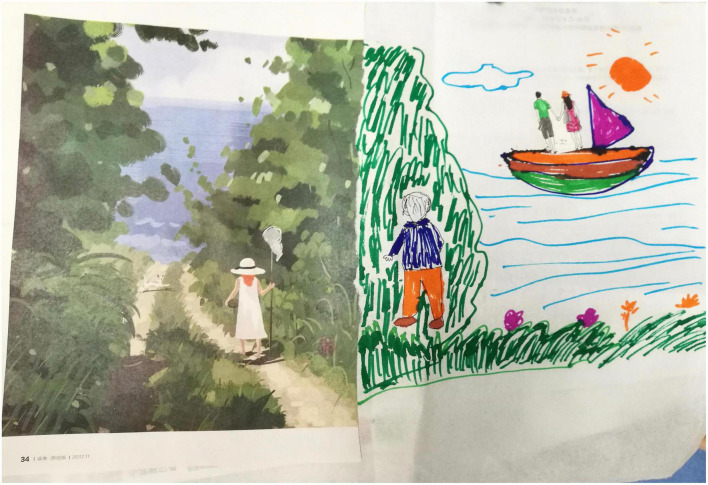
A painting showing the perception of pregnancy and childbirth.

The interviews were conducted by an experienced researcher who had received training in interviewing. An innovative method involving drawing was used during the interviews. It was considered a rich and insightful research method for exploring how people make sense of their world and was widely used on vulnerable and sensitive topics (Liamputtong and Suwankhong, 2015). All participants were invited to draw to match their real thoughts, emotions, and experiences, with a pack of 24 colored pens and a blank A3 sheet of paper. We also provided a number of magazines with a wealth of images to inspire participants. The whole drawing process was usually controlled at 30∼40 minutes. Before the drawing started, they would be informed that the main content of this drawing was to show their experience during pregnancy and their feelings about the upcoming childbirth. When they had finished, we asked the participants to describe their experience and feelings during pregnancy based on their drawing (see Figures 1, 2).

The process of the participant’s description of the drawing was combined with the interview process. That is, during the participants’ narration of the drawings, the researcher did not express subjective opinions, but asked questions at appropriate times in order to follow up and probe deeper into some issues in the scenario and facilitate the participants to express their true inner feelings. The researcher’s question was conducted in two ways: (1) the researcher asked questions based on some phenomena or issues mentioned in the participants’ narratives about the drawing; (2) the researcher asked questions at the right time based on open-ended questions prepared in advance (see Table 1). The entire interview was kept to 40∼60 minutes. All interviews were audio-recorded and then transcribed within 24 hours after the interviews were completed.

**TABLE 1:** Interview questions.

1. How about your experience during pregnancy? And how do you feel about the upcoming childbirth? (Instruction question before drawing)
2. Are you afraid of the childbirth? If so, what exactly are you afraid of?
3. Which delivery model would you take? Why?
4. What kind of support would be most helpful to you?
5. Would you like to share any other information that has not been mentioned?

### Data analysis

In this study, the data analysis method of Colaizzi (Morrow et al., 2015) was used to store, code, retrieve, and analyze the collected data with Nvivo11: (1) reading the interview transcripts in detail; (2) annotating statements of significance; (3) forming free nodes from recurring meaningful ideas; (4) analyzing the relationship between the free nodes and forming similar free nodes into tree nodes; (5) analyzing the tree nodes and describing them in detail; (6) sublimating thematic concepts; and (7) returning to the interviewees for confirmation (Morrow et al., 2015).

Two researchers simultaneously transcribed and organized the original data, independently coded, categorized, and refined the themes, and if they could not agree, the group discussed and reached a consensus to ensure the objectivity of the data analysis. Finally, the distilled themes were returned to the interviewees for validation.

### Ethic

This study was approved by the Ethics Committee of local University. The principle of informed consent was followed and a detailed and clear explanation of the purpose and process of the study was given before the interview.

## Results

A total of 20 participants, numbered P1 to P20, were included in this study. 14 participants were primiparous and 6 were multiparas, aged between 26 and 42 years (median = 30 years) and between 24 and 40 weeks of gestation (median = 29 weeks). Of these, 16 planned natural childbirth and 4 chose to have a cesarean section. The characteristics of the participants are shown in Table 2.

**TABLE 2:** Interview information and characteristics of participants.

Participants	Age (years)	Educational level	Occupation before pregnancy	Gestational weeks	Parity	Planned delivery methods
P1	29	Undergraduate	Unemployed	30	Primiparous	Vaginal delivery
P2	33	Undergraduate	Civil servant	28	Primiparous	Vaginal delivery
P3	42	Junior high school	Freelancer	25	Multipara	Cesarean section
P4	28	Undergraduate	Teacher	26	Primiparous	Cesarean section
P5	33	Senior high school	Freelancer	29	Multipara	Vaginal delivery
P6	28	Junior high school	Unemployed	33	Primiparous	Vaginal delivery
P7	31	Senior high school	Unemployed	38	Primiparous	Cesarean section
P8	35	Undergraduate	Teacher	40	Primiparous	Vaginal delivery
P9	35	Junior high school	Freelancer	33	Multipara	Cesarean section
P10	30	Undergraduate	Self-employer	40	Multipara	Vaginal delivery
P11	31	Undergraduate	Unemployed	36	Primiparous	Vaginal delivery
P12	32	Senior high school	Freelancer	28	Multipara	Vaginal delivery
P13	26	Undergraduate	Nurse	38	Primiparous	Vaginal delivery
P14	29	Undergraduate	Self-employer	27	Primiparous	Vaginal delivery
P15	30	Undergraduate	Salesman	24	Multipara	Vaginal delivery
P16	28	Undergraduate	Nurse	29	Primiparous	Vaginal delivery
P17	32	Undergraduate	Unemployed	29	Primiparous	Vaginal delivery
P18	27	Undergraduate	Unemployed	35	Primiparous	Vaginal delivery
P19	30	Undergraduate	Guided tour	33	Primiparous	Vaginal delivery
P20	33	Undergraduate	Management consultant	29	Primiparous	Vaginal delivery

Careful analysis of the interview transcripts and reference to the interview outline led to the use of class analysis to summarize the following 3 themes: “An invisible dilemma: trapped in lingering fear,” “An unexpected decision: choose to give birth naturally” and A strength to confront challenges head-on: move forward with fear.”

### An invisible dilemma: Trapped in lingering fear

Pregnant women were full of worries and fears, but others couldn’t understand. This made them feel deeply lonely and often struggle with contradictions alone.

#### Fear of all sides

The most common fear of birth described by participants was a concern for the health of the fetus, which they valued more than themselves.

“My early pregnancy reaction was very heavy, so I often worried about something terrible would happen, like abortion, fetal arrest, or fetal malformation” (P5)

Many women also mentioned concerns about the birth process. Notably, primiparas in this study mostly worried about their inability or loss of control during their childbirth. Multiparas were more afraid about childbirth pain. Additionally, some participants expressed their fears about obstetric interventions, or worried about the unfamiliar labor environment.

“This was my first time having a baby, and although I had watched a lot of videos and read books related to childbirth, I was still scared about going into labor because I had no idea what was going to happen to me” (P11).

“I have to go into the delivery room alone. I have no idea how I am going to get through that difficult time alone. It’s scary to think about” (P13).

Changes after giving birth were also under some participants’ concern, such as losing their partner’s attention, or being criticized and blamed for being unable to care for the child. In addition, two pregnant women expressed their fears about losing jobs and family economic status.

“I’m also worried that after childbirth I’m going to switch to my mother’s role and feel like I’m not mature enough to be a qualified mother” (P3).

“I have resigned. I have no choice but to look for it again in the future. I lost a lot of things because of pregnancy” (P15).

#### The untold loneliness

Participants also reported a sense of loneliness. It is due to lack of understanding, support, and effective communication with others, and also because pregnant women engage in self-isolation. Some participants reported that their fears of childbirth are not sufficiently understood and valued.

“Sometimes I feel scared thinking about upcoming childbirth. Although my husband would reassure me that it was okay, I didn’t think he really understood how I felt about it” (P6).

At the same time, some women engage in self-isolation and avoid discussing their fears about childbirth with others. Some participants did so because they felt that no one could help them and therefore preferred to remain silent. Two pregnant women felt it was inappropriate to fear childbirth as it should be a pleasant process traditionally.

“What’s the point of talking about it [fear of birth]? You still have to give birth and no one else can help you” (P18).

“It’s difficult to talk to my family about the fear of giving birth. Everyone is so pleased that I’m pregnant and I don’t want to break the good mood” (P7).

Lack of understanding from others and self-isolation create a vicious cycle. Some pregnant women reported that when they tried to communicate, negative reactions from others, such as lack of understanding, neglect, and perfunctory treatment, also acted as barriers to their communication.

“I think there’s no need to say anything. They can’t understand it. My fears were nothing to them. If you say too much, others think you’re very hypocritical” (P14).

#### Ambivalence with mixed feelings

Participants were in a state of ambivalence toward childbirth. Some participants described their fear of childbirth grew stronger as the birth date approaches, accompanied by anxiety, insomnia and other physical and mental symptoms. They wish the delivery day would come slower, and they try to find ways to escape the fear of childbirth.

“Now that I am getting closer to the expected delivery date. I sometimes cried when I thought about it. As much as I want to see my baby, I really wish time would pass more slowly” (P11).

At the same time, they also wished the time would pass faster due to the expectation of the baby’s arrival and being free from the bondage of pregnancy. Some women described there was a powerful inner drive that helped them to actively face and overcome their fears.

“I know it’s painful to give birth, but I just think it’s right. The baby will make everything I’ve experienced worth it” (P13).

### An unexpected decision: Choose to give birth naturally

Surrounded by fear, they made the unexpected choice to have a natural childbirth. Of the 20 participants, only four planned to have a cesarean section for physical reasons. The rest of the mothers all chose to have normal childbirth. This may be women’s initiative choice due to a strong belief in natural childbirth, or the result of passive acceptance.

#### Initiative selection

Participants believed that natural childbirth was the best way of birth, which was an important prerequisite for their preference for natural childbirth. Some women were passionate advocates of vaginal birth as they believed that childbirth should be a normal and natural process.

“I want to give birth naturally as much as possible, because it’s good for me and my baby” (P1).

Some women firmly chose to have natural childbirth due to a strong sense of responsibility to become good mothers. They thought it was conducive to the infants’ health.

“I wanted to do even things that were only a little good for my baby. So, I tended to be more inclined to have a natural birth. This might be my psychological growth in the process of nine months of pregnancy” (P8).

Encouragement and support from medical staff and family members will boost a woman’s belief and confidence in natural childbirth. And the medical staff provided enough information about the pros and cons of natural childbirth and cesarean section, and natural childbirth process which motivated pregnant women to eventually choose natural childbirth. In addition, their friends’ positive natural childbirth experience also helped women to establish a positive attitude and strengthen their belief toward natural childbirth.

“I was not firm about spontaneous labor at first. My husband has always been by my side, encouraged me, believed me, so can I insist on it” (P17).

“I heard from my friend that vaginal delivery only causes pain during childbirth and is very relaxed postpartum, unlike cesarean section” (P8).

It is worth noting that among the four pregnant women who planned to have a cesarean section for physical reasons, two of them showed their expectation of natural childbirth, despite knowing that their condition required a cesarean section.

“I actually want to ask if it’s possible to have a normal childbirth in my case [twin pregnancy] or not. I’ve read a lot of information before that it’s better to have a cesarean section, and the doctor also advised me to have a cesarean section, but I still want to have another consultation” (P4).

#### Passive acceptance

Some women described that they chose natural childbirth were influenced by significant others, including medical staff and family members, even though they themselves did not have a particularly strong desire for it. Many women stated that their doctor’s advice had a strong influence on their choice of birth method. If their doctor suggested them to have a normal birth, they were inclined to accept his or her advice out of authority and trust in him or her.

“The doctor said my condition was very suitable for vaginal birth and also gave me advice that vaginal birth was not traumatic to my body, so I accepted the doctor’s advice to have a trial vaginal childbirth” (P12).

Many women also described that family members played an important role in choosing delivery mode. Family members’ expectations of natural childbirth affected women’s final choice of delivery mode.

“My husband and mother-in-law wanted me to have a normal birth, even though I was scared. Maybe it’s the right choice” (P14).

Besides, some planning to have another child also should have natural childbirth plan as the context of China’s two-child policy. Because the cesarean section meant that there would be a long waiting time for another childbirth and a high probability of cesarean section again.

“If I can give birth naturally this time, I can have a child within one year. But if I have a cesarean section this time, I should wait at least three years to have a second child and might still have to have a cesarean section” (P20).

### A strength to confront challenges head-on: Move forward with fear

Once the decision has been made to have a natural birth, awaken of maternal spirit, hope in bloom, obstetric analgesia, and tolerance helped them to relief and overcome their fears of childbirth.

#### Awaken of maternal spirit

Many pregnant women described themselves realizing awaken of maternal spirit during pregnancy, which strengthened their belief in natural childbirth and gave them the courage to overcome their fears. The maternal spirit was mainly reflected in the strengthening of their role as mothers. With a deeper and closer tie established between them and their babies, they had a strong sense of responsibility to become good mothers. This made them change their mind from the initial desire for a cesarean section to a preference for a natural birth, as it was considered to be better for the baby’s health. Some women believed that their courage of motherhood would overcome fear of birth.

“At first, I wanted to choose the cesarean section because of my fear of labor pain. But my doctor and friends all advised me to give birth naturally. I know natural childbirth is good for both myself and my children, and now painless childbirth, so I changed my mind” (P11).

#### Hope in bloom

The participants described that they felt a sense of hope during their pregnancies. They shifted their fear of birth into the expectation of a better future after childbirth, which supported them in facing and overcoming the challenges of childbirth and fear. Besides, some of them regarded the baby as their hope and felt that the future deserved to look forward to.

“After childbirth, I will live a very happy and joyful life, so this [Fear] could be overcome” (P8).

“Although I still fear about the labor pain, I really look forward to having a baby. I believe that the courage to be a mother can overcome the feeling of fear. Therefore, if conditions permit, I will definitely choose natural childbirth, because it is good for the baby and mother” (P2).

#### Chinese tolerance culture

There are also some participants who choose to have a normal birth since they feel they should be tolerant of the labor pain for their baby’s health. Some women regarded enduring the p1ain of childbirth as one of the basic experiences that make a woman a good mother.

“Natural childbirth seems to be regarded as a particularly brave and great thing in today’s society. People around me think I’m very powerful when they know I want to have a natural childbirth. For the sake of the baby, although I don’t know how much my own pain tolerance is, what I think is that even if it hurts again, I should bear it and insist on a natural birth” (P8).

#### Obstetric analgesia

Many of the women in this study highlighted the importance of obstetric analgesia for their choice of natural childbirth, as this technique both eased their fears of labor pain and ensured a natural birth. They showed a strong willingness to use obstetric analgesia. One woman even described it as “light to humanity.”

“I must give birth with labor analgesia. I would not have a cesarean section, or give birth without labor analgesia. Otherwise, I can’t complete the labor” (P19).

“I was afraid of pain. After I knew that painless labor could be used, my mood was relieved” (P18).

The fear of birth was shifted into concern about the uncertainty of using obstetric analgesia. One woman even decided to give birth in another city to make sure of using obstetric analgesia.

“I have seen lots of cases on the Internet where women cannot use obstetric analgesia in hospitals due to a lack of doctors. I am really worried that this will happen to me, so even if this hospital is far from me, I will come here to give birth” (P20).

Notably, no women in this study show any concerns about the side effects of obstetric analgesia.

## Discussion

This study aimed to investigate Chinese women’s experience of childbirth fear and their preference for the delivery mode. The results showed that fear of birth was a synthesis of multiple complex emotions. In addition to the fear, women with the fear of birth also experienced a sense of loneliness and ambivalence. Also, we found that participants in this study preferred vaginal birth, actively choose or passively accept it. After women decided to have a normal birth, they overcame and eased their fears of childbirth through internal factors including awaken of maternal spirit, hope in bloom and Chinese tolerance culture, and external factors of obstetric analgesia.

Participants with a fear of birth experienced complex feelings formed by three moods: fear, loneliness, and ambivalence. Previous research has mainly focused on fear (Serçekuş and Okumuş, 2009; Karlsdottir et al., 2015), while the other two have rarely been reported. In this study, women’s greatest fear was the baby’s health, followed by fear of labor pain and loss of control, and fear of medical interventions and the hospital environment. The result is supported by numerous previous research (Serçekuş and Okumuş, 2009; Rilby et al., 2012; Karlsdottir et al., 2015; Takegata et al., 2018). Loneliness was also reported to come with the fear of birth. Cultural background might play an important role. Childbirth was considered to be happy in Chinese culture, which made some women feel ashamed of their fear. Besides, the Chinese’s traditional and introverted personalities might have exacerbated their self-enclosed and finally resulted in a sense of loneliness. Nilsson’s (Nilsson and Lundgren, 2009) study in Sweden also reported communication barriers of women with childbirth fear. This might be due to the similar cultural context and environment, where women’s fear of birth was not respected. In addition, a sense of ambivalence in perceptions of childbirth and the choice of delivery mode was reported. Obstetric medical staff should therefore comprehensively understand the complexities of the fear of birth and respect its existence, strengthen the relationship with pregnant women and encourage their emotional expression to alleviate their sense of fear, loneliness and ambivalence.

Surprisingly, we found that 16 out of 20 participants with the fear of birth in this study had vaginal birth plans. This is inconsistent with previous studies in which women with childbirth fear led to an increased rate of selective cesarean section. In this study, women’s choice to have a normal birth may be an active or passive choice. For women who actively chose vaginal childbirth in this study, their attitudes that natural birth was a better way to give birth are a fundamental premise for the choice of birth mode. This is in line with an Iran study, in which participants believe the superiority of vaginal delivery, due to its positive outcomes for both mother and infant (Zakerihamidi et al., 2015). At the same time, the support of significant others will strengthen their belief and confidence in natural childbirth. Bandura believes that the alternative experience provided by important others, as well as their trust and support for personal abilities, can enhance the confidence and self-efficacy of childbirth (Bandura and Watts, 1995). Meanwhile, adequate social support can also help pregnant women acquire knowledge and reduce stress.

The impact of important others is not only in their support for pregnant women. Women who lack belief in natural childbirth may be forced to choose a normal birth because of their doctors’ advice or the family’s expectations. Previous studies have shown that women are vulnerable to family, peers, and other close people. Our results are consistent with these studies. In China, medical personnel often dominate and are regarded as an authority, so their suggestions have a great impact on women’s choice of delivery mode. In addition, influenced by Chinese traditional culture that stressed the concept of “more children, more blessings”, many families believed that two children would be more conducive to family harmony and children’s growth. Women are thus more inclined to have a second child in the context of the two-child policy, and thus choose natural childbirth to shorter the interval between two children and ensure the safety of the child (Johnson, 2018). Therefore, obstetric medical staff should strengthen health education for pregnant women, help them correctly understand the advantages and disadvantages of natural childbirth and cesarean section and correct misunderstanding, reduce the doubt and fear of natural childbirth. In addition, we should fully mobilize the social support system for pregnant women, and strengthen the care and support for pregnant women, to eliminate their loneliness, helplessness, and fear.

Once women have decided to have a normal birth, they need to overcome their fear of birth. It has been shown that overcoming fear is a result of the combined action of intrinsic and extrinsic factors. Intrinsic motivation consists in the awakening of the maternal spirit, a sense of hope and tolerance. It is worth noting that there is a paucity of research on this topic. Many women described themselves as realizing that the awakening of the maternal spirit, the process of adapting to motherhood, and the closer mother-infant relationship supported them in overcoming their fear of birth. Our findings are supported by many other studies. Research evidence suggested that the transition to motherhood from early pregnancy to birth was an important factor in a mother’s ability to cope, overcome problems during the process, and adapt to the new environment (Coşkuner Potur et al., 2017). Also, a systematic review revealed that a closer mother-infant relationship could enhance women’s resilience and have a positive impact on their psychological well-being (Rouhe et al., 2015).

Among the internal factors, the role of hope in choosing a delivery mode was also reported, which was unprecedented in previous studies. According to Hope Theory, Hope helps people articulate their goals and their ability to develop and implement specific strategies to achieve them (Rand and Cheavens, 2009). Various studies have shown that high levels of hope are associated with physical and mental health, high self-esteem, and positive thinking. So, a sense of hope in women with birth fears helped them to feel more positive and motivated to break through the fear of birth and achieve their goals of giving birth naturally to a healthy baby (Hoseini et al., 2020). Notably, research has shown a significant relationship between the adequacy of prenatal care and hope scores (Hoseini et al., 2020), suggesting that obstetricians can help women overcome their childbirth fear by providing adequate care to increase their sense of hope.

In this study, “Tolerance” of Chinese traditional culture was also used to cope with the fear of birth. It is an important and commonly prevalent psychosocial phenomenon that potentially influences Chinese people’s behavior. The thought of tolerance is a strategic self-repression and self-adjustment cognitive process closely related to the individual’s attitude towards the object to be tolerated. When women identify with and decide to have a natural birth, this personality trait helps them to proactively shift their attention and cognitive changes to overcome or suppress their inner fears and achieve their desired goal. However, it is important to note that endurance could bring an unpleasant emotional experience. Health professionals should help women to confront their emotions rationally, without prejudice, and consciously, rather than by ‘suppressing’ them.

Research has shown that obstetric analgesia is a very important external factor in helping women overcome their fear of birth. In this study, obstetric analgesia, as a breakthrough between natural birth and fear of birth, played a very important role in the choice of delivery mode. Studies have shown that obstetric analgesia increases the incidence of spontaneous birth (Hul et al., 2015). However, we found that some participants showed fanatical expectations and excessive dependence on obstetric analgesia. They transformed concerns about pain into the uncertainty of obstetric analgesia, which may be another sign of avoidance of labor and fear of birth. This is at variance with previous research, which found in a Japanese study that pregnant women were more cautious about choosing obstetric analgesia because they felt that experiencing labor pain was an important part of motherhood and felt guilty for choosing it (Takegata et al., 2018). At present, obstetric analgesia technology in China is not mature, the scope of use is not broad, and related education is not sufficient. We should be vigilant of the possibility of a backlash when painless childbirth cannot be achieved, resulting in a negative birth experience or even birth trauma. Therefore, we should focus on and improve these deficiencies of obstetric analgesia in China in the future, and strengthen health education interventions on non-pharmacological methods of analgesia to avoid the adverse effects of blindly choosing obstetric analgesia.

Several limitations to our study should be considered. Firstly, this study was conducted only in a tertiary hospital located in central China, and the Chinese cultural context played an important role in participants’ experience and feelings, the transferability of the findings thus needs further validation. Secondly, we only interviewed each participant once and failed to capture their experiences over time. Therefore, more longitudinal studies are needed to fully understand women’s experiences of fear of birth in different regions and across different trimesters. Thirdly, for the six individuals interviewed after the pandemic, we did not fully consider the potential impact of the post-pandemic psychological consequences on their fear of childbirth.

## Conclusion

In conclusion, fear of birth is a complex emotion, accompanied by feelings of loneliness and ambivalence in addition to fear. We found that women with fear of birth in this study prefer vaginal birth, and it was revealed to be the result of a combined action of intrinsic and extrinsic factors. Healthcare providers should provide adequate professional support during antenatal care, and fully mobilize the social support system for pregnant women. So as to, on the one hand, eliminate their sense of loneliness, helplessness, and fear, and at the same time can stimulate intrinsic motivation, awaken the maternal strength, and increase their sense of hope, so that to face their fear of birth with courage. In addition, a more cautious attitude should be taken towards obstetric analgesia, improving the deficiencies of obstetric analgesia in China and strengthening health education interventions to avoid the adverse effects of blindly choosing obstetric analgesia.

## Data availability statement

The original contributions presented in this study are included in the article/supplementary material, further inquiries can be directed to the corresponding author.

## Ethics statement

The studies involving human participants were reviewed and approved by the Ethics Committee of Tongji Hospital, Tongji Medical College, and Huazhong University of Science and technology. The patients/participants provided their written informed consent to participate in this study. Written informed consent was obtained from the individual(s) for the publication of any potentially identifiable images or data included in this article.

## Author contributions

TZ: conceptualization, data curation, writing—review and editing, and supervision. MY: conceptualization, investigation, data curation, roles/writing—original draft, and formal analysis. MW: investigation, data curation, and writing — review and editing. YC: data curation and writing — review and editing. KZ: investigation and data curation. All authors contributed to the article and approved the submitted version.
